# Radical Scavenging and Cellular Antioxidant Activity of the Cocoa Shell Phenolic Compounds after Simulated Digestion

**DOI:** 10.3390/antiox12051007

**Published:** 2023-04-26

**Authors:** Silvia Cañas, Miguel Rebollo-Hernanz, Patricia Bermúdez-Gómez, Pilar Rodríguez-Rodríguez, Cheyenne Braojos, Alicia Gil-Ramírez, Vanesa Benítez, Yolanda Aguilera, María A. Martín-Cabrejas

**Affiliations:** 1 Department of Agricultural Chemistry and Food Science, Faculty of Science, C/Francisco Tomás y Valiente, 7, Universidad Autónoma de Madrid, 28049 Madrid, Spain; 2 Institute of Food Science Research (CIAL, UAM-CSIC), C/Nicolás Cabrera, 9, Universidad Autónoma de Madrid, 28049 Madrid, Spain; 3 Department of Physiology, Faculty of Medicine, Universidad Autónoma de Madrid, C/Arzobispo Morcillo 2, 28029 Madrid, Spain

**Keywords:** cocoa shell, cocoa by-products, in vitro digestion, phenolic compounds, oxidative stress, antioxidant capacity, radical scavenging, reactive oxygen species, antioxidant enzymes

## Abstract

The cocoa industry generates a considerable quantity of cocoa shell, a by-product with high levels of methylxanthines and phenolic compounds. Nevertheless, the digestion process can extensively modify these compounds’ bioaccessibility, bioavailability, and bioactivity as a consequence of their transformation. Hence, this work’s objective was to assess the influence of simulated gastrointestinal digestion on the concentration of phenolic compounds found in the cocoa shell flour (CSF) and the cocoa shell extract (CSE), as well as to investigate their radical scavenging capacity and antioxidant activity in both intestinal epithelial (IEC-6) and hepatic (HepG2) cells. The CSF and the CSE exhibited a high amount of methylxanthines (theobromine and caffeine) and phenolic compounds, mainly gallic acid and (+)-catechin, which persisted through the course of the simulated digestion. Gastrointestinal digestion increased the antioxidant capacity of the CSF and the CSE, which also displayed free radical scavenging capacity during the simulated digestion. Neither the CSF nor the CSE exhibited cytotoxicity in intestinal epithelial (IEC-6) or hepatic (HepG2) cells. Moreover, they effectively counteracted oxidative stress triggered by *tert*-butyl hydroperoxide (*t*-BHP) while preventing the decline of glutathione, thiol groups, superoxide dismutase, and catalase activities in both cell lines. Our study suggests that the cocoa shell may serve as a functional food ingredient for promoting health, owing to its rich concentration of antioxidant compounds that could support combating the cellular oxidative stress associated with chronic disease development.

## 1. Introduction

Cocoa is an important crop worldwide, predominantly grown in tropical areas, and recognized as the primary raw material source for chocolate manufacturing [1]. Global cocoa production is approximately 5.0 million tons annually [2]. A large quantity of by-products are discarded during cocoa processing, including the cocoa shell (CS), which accounts for 12–20% of the cocoa seed [3]. The CS is composed mainly of carbohydrates (8–26%), proteins (12–22%), lipids (2–18%), and dietary fiber (40–64%), highlighting its insoluble fraction (24–52%). Furthermore, the CS also comprises different bioactives, including phenolic compounds and methylxanthines (theobromine and caffeine) [4]. Nowadays, interest in phenolic compounds and other antioxidant phytochemicals has increased because their intake may contribute to reducing oxidative stress [5].

Reactive oxygen species (ROS) and reactive nitrogen species (RNS) are produced during normal cellular metabolism and exposure to environmental stressors. ROS and RNS encompass a group of highly reactive molecules, including but not limited to superoxide anion radical (O_2_^•−^), hydroxyl radical (^•^OH), and hydrogen peroxide (H_2_O_2_), as well as reactive nitrogen species such as nitric oxide (NO^•^) and peroxynitrite (ONOO^−^). These species induce oxidative and nitrosative stress, produced as a consequence of the imbalance between oxidative and antioxidant species in favor of oxidants, which leads to cellular damage and dysfunction [6]. These compounds can damage cells and contribute to the onset of various diseases, including diabetes, cancer, hypertension, and neurological and cardiovascular disorders [7]. Phenolic compounds contribute to preventing these diseases by diminishing oxidative stress and offering protection against cellular damage [8]. Research indicates that phenolic compounds may synergize with other exogenous antioxidants, such as vitamins C and E, to deliver enhanced protection [9]. Furthermore, phenolic compounds have been shown to increase the body’s endogenous antioxidant defenses, including enzymes such as superoxide dismutase (SOD), catalase (CAT), glutathione peroxidase (GPx), and glutathione reductase (GR) [10].

Our previous investigations have demonstrated that the CS exhibits a significant antioxidant capacity, primarily attributed to a defined phenolic compound profile and other antioxidant constituents [11]. This antioxidant activity has been observed in vitro and ex vivo and has been associated with the prevention of mitochondrial dysfunction [12], insulin resistance [13], endothelial dysfunction [14], and metabolic syndrome [15]. In order to exert their biological properties, phenolic compounds must be absorbed in the gastrointestinal tract. Hence, the health-promoting effects of phenolic compounds are largely determined by their bioaccessibility and bioavailability [16]. Recently, our research group has studied the bioaccessibility and gastrointestinal fate of the CS’s phenolic compounds using the harmonized simulated digestion model INFOGEST and their bioavailability in silico. Although gastrointestinal digestion can cause the degradation and transformation of certain phenolic compounds, a significant proportion of these compounds remain intact and bioaccessible [17]. In addition to determining phytochemicals’ bioaccessibility and bioavailability, assessing their antioxidant activity using assays that reflect physiological conditions is also essential. Prior to animal studies and human clinical trials, cell culture models are utilized for preliminary antioxidant screening in foods, as they allow rapid and economic analyses [18]. Given that digestion and nutrient absorption take place in the intestine, which also serves as a physical barrier against foreign substances and pathogens, and considering the liver’s role in nutrient metabolism, these tissues are more susceptible to oxidative stress and stress conditions compared to other organs [19,20]. Hence, the study of oxidative stress in intestinal and liver cells is crucial. The goal of this study was to investigate the influence of simulated digestion on the concentration of phenolic compounds in the flour (CSF) and an aqueous extract (CSE) from the CS and the implications for in vitro radical scavenging capacity and antioxidant activity in hepatic and intestinal cells.

## 2. Materials and Methods

### 2.1. Materials

Acetonitrile, ethylenediaminetetraacetic acid (EDTA), ferric chloride hexahydrate, Folin–Ciocalteu reagent, formic acid, hydrochloride acid, methanol, nitric acid, potassium chloride, sodium bicarbonate, sodium carbonate, sodium chloride, sodium hydroxide, sodium nitrite, and sodium nitroprusside were provided by Panreac Química S.L.U. (Barcelona, Spain). Reference phytochemicals (purity ≥ 96%), including 1,3,7-trimethylxanthine, 3,3′,4′,5,7-pentahydroxyflavone 3-β-glucoside, 3,4,5-trihydroxybenzoic acid, 3,4-dihydroxybenzoic acid, 3′,4′-dihydroxycinnamic acid, 3,7-dimethylxanthine, 4-hydroxy-3-methoxybenzoic acid, 4′-hydroxycinnamic acid, and 4′,5,7-trihydroxyflavone-6-C-glucoside were acquired from Sigma-Aldrich (Sigma-Aldrich, Alcobendas, Spain) and Extrasynthese (Genay, France). The 2,2′-Azino-bis(3-ethylbenzothiazoline-6-sulfonic acid) (ABTS), 2,4,6-tris(2-pyridyl)-s-triazine (TPTZ), 2′,7′-dichlorofluorescin diacetate (DCFDA), 5,5-dithiobis-2-nitrobenzoic acid (DTNB), 6-hydroxy-2,5,7,8-tetramethylchromane-2-carboxylic acid (Trolox), *o*-phthalaldehyde (OPT), ammonium carbonate, ammonium hydrogen carbonate, ascorbic acid, calcium chloride dihydrate, Griess reagent, hydrogen peroxide, magnesium chloride hexahydrate, manganese oxide, nitrotetrazolium blue chloride (NBT), porcine pancreatin, porcine pepsin, potassium persulfate, potassium phosphate monobasic, pronase E, pyrogallol, pyrogallol red, reduced glutathione (GSH), *tert*-butyl hydroperoxide (*t*-BHP), and Viscozyme were provided by Sigma-Aldrich (Sigma-Aldrich, Alcobendas, Spain). 

### 2.2. CSF and CSE Processing

The CS was given by Chocolates Santocildes (Castrocontrigo, León, Spain). For obtaining the CSF, the CS was milled and stored in closed flasks protected from light at −20 °C until use. The aqueous CSE was prepared following a previously optimized extraction protocol [11]. The CSF was boiled in water (100 °C, 0.02 g mL^−1^ flour-to-water ratio) and agitated for 90 min. The aqueous CSE obtained required filtration, freezing at −20 °C, and freeze-drying to be finally prepared. Similarly, the CSE was stored in closed flasks protected from light at −20 °C until further use.

### 2.3. CSF and CSE In Vitro Gastrointestinal Digestion

The in vitro simulated static digestion of the CSF and the CSE was carried out following the INFOGEST procedure with minor adjustments [21]. In brief, the CSF (1 g) or the freeze-dried CSE (0.1 g) were combined with a simulated oral fluid and stirred for 2 min at 37 °C to mimic the oral fraction. Due to the lack of starch in the samples, no amylase was added. To complete the gastric phase, the resulting oral phase was merged with the simulated gastric fluid and pepsin (2000 U mL^−1^) and incubated under continuous stirring (2 h, 37 °C). To model the intestinal phase, the gastric phase was combined with the simulated intestinal fluid that included pancreatin (100 U trypsin mL^−1^) and incubated for 2 h at 37 °C. The colonic phase was performed as previously described [22]. The intestinal phase was mixed with 5 mL of Pronase E (1 mg mL^−1^), the pH was adjusted to 8.0, and the digested CS was stirred at 37 °C for 1 h. After that, the pH in the digestion media was adjusted to 4. Viscozyme (150 µL, 0.08 U μL^−1^) was added, and the colonic digestion media were further incubated under stirring (37 °C, 16 h). All digestions were performed in duplicate. An empty digestion blank was run for each digestion phase, comprising the mixture of enzymes and reagents found in the simulated digestion fluids. The bioaccessible (supernatants) and non-digested fractions (residues) gathered from each digestion phase were freeze-dried and stored at −20 °C until utilization.

### 2.4. Phytochemical Profile Analysis by HPLC-PDA-ESI/MSn

The analysis of phenolic compounds and methylxanthines in the bioaccessible digestion fractions from the CS was performed following a previously described methodology [17]. The CS’s phytochemicals were separated using an HPLC coupled with a photodiode array detector (PDA) (Hewlett-Packard-1100, Agilent Technologies, Palo Alto, CA, USA) using a Spherisorb S3 ODS-2 C8 (3 µm, 150 mm × 4.6 mm i.d.) column from Waters (Milford, MA, USA). The elution gradient was achieved through a stepwise increase in solvent B concentration: 15% for 5 min, 15–20% for 5 min, 20–25% for 10 min, 25–35% for 10 min, and 35–50% for 10 min, followed by column re-equilibration. The mobile phases were 0.1% formic acid in water (solvent A) and 100% acetonitrile (solvent B), and the flow rate was set to 0.5 mL min^−1^ at 35 °C. The concentration of each compound was expressed as µg 100 g^−1^ of the digested CS. The potential bioaccessibility index was calculated as (1):

(1) $$\mathrm{Potential}\;\mathrm{bioaccesibility}\;\mathrm{index}=\frac{C_{GI}}{C_{ND}}$$

where C_GI_ is the indicated bioactive compound content after simulated gastrointestinal digestion and C_ND_ is the same bioactive compound content in CSF or CSE before digestion.

### 2.5. Extraction of Free and Bound Phenolic Compounds

The extraction of free and bound phenolic compounds was performed as previously detailed [23]. Free phenolic compounds were extracted from the non-digested CSF fractions obtained after the in vitro digestion of the CSF using acidified (0.1% HCl) methanol/water (80:20, *v*/*v*). The residues from the previous step were hydrolyzed in an alkali medium (4 mol L^−1^ NaOH), and bound phenolic compounds were obtained from the hydrolyzed non-digested fractions.

### 2.6. Assessment of the Total Phenolic Content

The total phenolic content (TPC) was determined using the Folin–Ciocalteu colorimetric method [24]. As previously described, the experiment was adapted to a micromethod format in 96-well plates [25]. The TPC values were reported as mg 3,4,5-trihydroxybenzoic (gallic) acid equivalents per gram (mg GAE g^−1^).

### 2.7. Evaluation of the In Vitro Antioxidant Capacity 

#### 2.7.1. ABTS Antioxidant Capacity

The antioxidant capacity was estimated by the ABTS method, as reported before [23]. To obtain ABTS˙^+^ radical cations, 7 mmol L^−1^ ABTS was combined with 2.45 mmol L^−1^ K_2_S_2_O_8_ and incubated at room temperature for 16 h under stirring, protected from light. The obtained ABTS˙^+^ solution was diluted to reach an optical density of 0.70 at 734 nm by diluting in 5 mmol L^−1^ PBS, pH 7.4. The assay was conducted in a 96-well plate by mixing 30 μL of the digested CS or standard with 270 μL of the diluted ABTS˙^+^ solution and letting it react for 10 min in the dark. Finally, the optical density was measured at 734 nm. Trolox (0–0.06 mg mL^−1^) served as the standard for preparing a calibration curve. The antioxidant capacity was calculated as mg Trolox equivalent per gram (mg TE g^−1^). 

#### 2.7.2. Ferric Reducing Antioxidant Power (FRAP)

The FRAP assay was performed following the already explained method [26]. FRAP reagent was produced by combining 0.3 mol L^−1^ acetate buffer pH 3.6 with 10 mmol L^−1^ TPTZ, 40 mmol L^−1^ HCl, and 20 mmol L^−1^ FeCl_3_·6H_2_O (10:1:1, *v*/*v*/*v*). The assay was performed by reacting 10 μL of the digested CS or standard and 300 μL of the FRAP solution in a 96-well plate and incubating at 37 °C for 10 min. Finally, the absorbance was recorded at 593 nm. Trolox (25–800 µmol L^−1^) served as the standard for preparing a calibration curve. The antioxidant capacity was expressed as mmol Trolox equivalent per gram (mmol TE g^−1^).

### 2.8. Evaluation of the ROS and RNS Scavenging Capacity

#### 2.8.1. Superoxide Anion Radical (O_2_^•−^) Scavenging Capacity

O_2_^•−^ scavenging was determined using a described protocol with slight modifications [27]. The method is based on the inhibition of the formation of blue diformazan resulting from the reduction of NBT by the superoxide generated from pyrogallol autoxidation. Briefly, 100 µL of the digested CS were combined with 100 µL of 50 mmol L^−1^ ammonium hydrogen carbonate buffer (pH 9.3, 0.33 mmol L^−1^ EDTA) in a 96-well plate. Then, 30 µL of NBT (50 µmol L^−1^) were added, and the mixture was agitated vigorously. Finally, 30 µL of pyrogallol (16.5 mmol L^−1^) were added, and the plate was shaken. Blanks were prepared for each sample. Ascorbic acid (0–70 mg mL^−1^) was used as a standard. The radical scavenging capacity was expressed as g ascorbic acid equivalent per gram (g AAE g^−1^).

#### 2.8.2. Hydrogen Peroxide (H_2_O_2_) Scavenging Capacity

The ability of the digested CS to scavenge H_2_O_2_ was analyzed according to Grancieri et al. [28], with slight modifications. The digested CS (120 µL) was combined with 70 µL of H_2_O_2_ (40 mmol L^−1^). The mix was stirred and incubated for 10 min at room temperature. The optical density was measured at 230 nm using a microplate reader. Blanks were prepared for each sample. Ascorbic acid (0–1250 µg mL^−1^) was used as a standard. The radical scavenging capacity was expressed as mg ascorbic acid equivalent per g (mg AAE g^−1^). 

#### 2.8.3. Nitric Oxide (NO) Scavenging Capacity 

The NO scavenging capacity was evaluated following the method described by Grancieri et al. [28], with slight modifications. The NO generated from sodium nitroprusside is decomposed in the presence of oxygen, generating nitrite (NO_2_), which can be detected spectrophotometrically by forming a magenta chromophore with the Griess reagent. Briefly, 50 µL of the digested CS were added to a 96-well plate and combined with 50 µL of sodium nitroprusside (20 mmol L^−1^). The plate was kept in agitation for 5 min at room temperature, and 50 µL of Griess reagent were added to each well. The optical density was recorded at 550 nm in a microplate reader. Blanks were prepared for each sample. A curve of ascorbic acid (0–2500 µg mL^−1^) was prepared. The radical scavenging capacity was expressed as mg ascorbic acid equivalent per g (mg AAE g^−1^). 

#### 2.8.4. Peroxynitrite (ONOO^−^) Scavenging Capacity 

ONOO^−^ was synthesized following the method of Robinson and Beckman with slight modifications [29]. 75 mL of acidified 2 mol L^−1^ H_2_O_2_ (2 mol L^−1^ HNO_3_) was mixed with 75 mL of NaNO_2_ (2 mol L^−1^) on an ice bath under vigorous stirring. The reaction was stopped after 1 s by adding 150 mL of NaOH (4 mol L^−1^). The yellow ONOO^−^ solution was kept on ice. The residual H_2_O_2_ was removed by adding MnO_2_ (7 mg mL^−1^) and stirring for 1 h. The solution was centrifuged for 5 min at 1600× *g*, and the supernatant obtained was filtered. The ONOO^−^ obtained was frozen at −80 °C until use, and its concentration was defined spectrophotometrically at 302 nm (ε = 1670 M^−1^ cm^−1^). 

The pyrogallol red bleaching assay was employed to quantify the scavenging of ONOO^−^ through competition kinetics [30]. Briefly, 70 µL of the digested CS were mixed with 140 µL of pyrogallol red (100 µmol L^−1^) in a 96-well plate. The mix was incubated under agitation for 15 min, and 40 µL of peroxynitrite (500 µmol L^−1^) were added. After 10 min, the optical density was measured spectrophotometrically at 540 nm. Blanks were prepared for each sample. Ascorbic acid was used as a standard (0–500 µg mL^−1^). The radical scavenging capacity was expressed as mg ascorbic acid equivalent per g (mg AAE g^−1^). 

### 2.9. Antioxidant Activity in Cell Culture-Based Experiments

Rat intestinal epithelial IEC-6 and human hepatoma HepG2 cell lines were acquired from the American Type Culture Collection (ATCC, Rockville, MD, USA). IEC-6 cells were grown in Dulbecco’s Modified Eagle Medium (DMEM), containing 4.5 g L^−1^ glucose, and supplemented with 10% fetal bovine serum (FBS), 1% L-glutamine, 1% penicillin/streptomycin, and 0.1 U mL^−1^ bovine insulin. HepG2 cells were cultured in DMEM containing FBS (10%), L-glutamine (1%), and penicillin/streptomycin (1%). Cell cultures were incubated at 37 °C and 100% humidity in a 5% CO_2_ atmosphere using a humidified incubator (BINDER CB series 2010, Tuttlingen, Germany).

#### 2.9.1. Cell Viability

To determine viability, IEC-6 and HepG2 cells were grown in 96-well plates at 5.0 × 10^5^ cells per mL. After 24 h, the cells were treated with the digested CSF and the CSE (200 μg mL^−1^) and incubated for 24 h. Cell viability was determined using the CellTiter^®^ 96 Aqueous One Solution Proliferation assay (Promega, Madison, WI, USA) as indicated by the manufacturer.

#### 2.9.2. Intracellular Reactive Oxygen Species (ROS) Measurement

IEC-6 and HepG2 were seeded into a 96-well culture plate (1.0 × 10^4^ cells per well) and incubated for 24 h. After incubation, cells were pre-treated with the digested extracts from CSF and CSE at 200 μg mL^−1^ for 24 h. Cells were rinsed with PBS and incubated with 12.5 µmol L^−1^ DCFDA (final concentration) for 40 min. After incubation, the cells were rewashed with PBS and co-treated with the digested CSF or CSE at 200 μg mL^−1^ in the presence of *t*-BHP (1 mmol L^−1^) for 1 h. Cells were ultimately washed with PBS, and ROS production was examined using a fluorescence reader. The signal was measured at excitation/emission wavelengths of 485/530 nm, respectively. Results were normalized against cell viability values. 

#### 2.9.3. Cell Lysates

To determine the levels of GSH, thiol groups, and the activities of CAT and SOD, IEC-6 and HepG2 cells were seeded into a 6-well culture plate (1.0 × 10^6^ cells per well). After 48 h of incubation, cells were washed with PBS and treated with non-digested and digested extracts from CSF and CSE at 200 μg mL^−1^ in the presence of 1 mmol L^−1^ *t*-BHP for 2 h (IEC-6), and 3 h (HepG2). Cells treated with different conditions were lysed at 4 °C in PBS by ultrasonication after removing the culture medium. The cells were then centrifuged at 1200× *g* for 30 min. Supernatants were collected, the protein concentration was determined using the Bradford reagent, and all lysates were aliquoted and stored at −80 °C until used for further analysis. 

#### 2.9.4. Reduced Glutathione (GSH)

Reduced glutathione was determined using a fluorometric assay by means of the OPT reaction [31]. In brief, 10 µL of each cell lysate were mixed with 12.5 µL of HPO_3_ (25% *w*/*v*) and 37 µL of phosphate-EDTA buffer, pH 8.0 (0.1 mol L^−1^ sodium phosphate in 5 mmol L^−1^ EDTA). The mixture was preserved on ice for 10 min and centrifuged at 10,000× *g* for 15 min at 4 °C. After centrifugation, 10 µL of each supernatant, 180 µL of phosphate-EDTA buffer, and 10 µL of OPT (0.1% *w*/*v* methanol) were added to a 96-well plate. The plate was shaken for 1 min and incubated at room temperature in the dark for 15 min. The fluorescence signal was analyzed at 360 nm excitation and 460 nm emission in a microplate spectrophotometer. Blanks were performed, adding 10 µL of each cell lysate and 190 µL of phosphate-EDTA buffer. A calibration curve of GSH (0–10 µg mL^−1^) was performed. The GSH level of the cell lysates was expressed as nmol GSH mg^−1^ protein.

#### 2.9.5. Thiol Groups 

The thiol group method is based on the ability of the thiol groups to react with Ellman’s reagent (DTNB) [32]. In brief, 10 µL of supernatants were combined with 200 µL of 0.5 mmol L^−1^ DTNB in phosphate-potassium-saline buffer, pH 7.4, in a 96-well plate. The plate was shaken and incubated for 30 min at room temperature in the dark. The optical density was read at 412 nm. Blanks were prepared by mixing 10 µL of each cell lysate with 200 µL of phosphate-potassium-saline buffer. A standard curve was prepared using pure GSH (0–0.5 mmol L^−1^). Thiol levels were expressed as mol GSH mg^−1^ protein.

#### 2.9.6. Catalase (CAT) and Superoxide Dismutase (SOD) Activity

CAT and SOD activities were assessed using commercial kits (KB-03-012 and KB-03-011, respectively, Bioquochem, Gijon, Spain), following the indication provided by the manufacturer. CAT and SOD activity were reported as U mg^−1^ protein.

### 2.10. Statistical Analysis

The results were presented as the mean ± standard deviation (SD) of at least three independent experiments (*n* = 3). Statistical analysis was performed using one-way analysis of variance (ANOVA), followed by a post hoc Tukey’s test, to compare the different digestive fractions. To compare against the non-treated control group (OX), which consisted of oxidized cells or non-treated cells (NT) in cell culture studies, a one-way ANOVA and post-hoc Dunnett’s test were conducted. Differences were considered significant at a *p* < 0.05. To account for positive and negative effects in the comparison and allow for the observation of the effects of the digestion fraction, the percentage of protection against oxidative damage in cell culture experiments was calculated using Equation (2):

(2) $$\%\;\mathrm{protection}=1-\frac{\left(NT-V_{i}\right)}{\left(NT-OX\right)}$$

where *NT* is the parameter value in non-treated cells, *OX* in cells stimulated with *t*-BHP, and *V_i_* is the parameter value in each digestion fraction [33]. Pearson correlations were calculated to examine the associations between the digested CSF and CSE phenolic profiles and their antioxidant properties. Principal component analysis (PCA) and hierarchical cluster analysis were used to classify the CS based on their phenolic composition and antioxidant properties. GraphPad Prism 8.0 (San Diego, CA, USA) was utilized for univariate and bivariate statistical analyses and to produce the bar graphs. XLSTAT2021 was employed to run multivariate analysis (PCA and hierarchical clustering).

## 3. Results and Discussion

### 3.1. The Cocoa Shell Contained a High Content of Phenolic Compounds and Methylxanthines Which Were Released during Digestion

Phenolic compounds were determined by HPLC-PDA-MSn in the CSF and the CSE (Table 1). The non-digested CSF was composed of hydroxybenzoic acids (22.6 mg 100 g^−1^), *N*-phenylpropenoyl-L-amino acids (7.4 mg 100 g^−1^), flavan-3-ols (12.8 mg 100 g^−1^), flavonols (0.6 mg 100 g^−1^), and a large amount of methylxanthines (695.2 mg 100 g^−1^). Hydroxybenzoic acids from the CSF were completely liberated at the end of the in vitro digestion. The 3,4,5-Trihydroxybenzoic (gallic) and 3,4-dihydroxybenzoic (protocatechuic) acids exhibited an intestinal bioaccessibility value of 0.7. Regarding the *N*-phenylpropenoyl-L-amino acids, *N*-(3-(3,4-dihydroxycinnamoyl)-L-aspartate, the primary compound in the non-digested CSF (5.1 mg 100 g^−1^), was not detected during digestion. The *cis* and *trans* isomers of *N*-(3-(4-hydroxycinnamoyl)-L-aspartate increased by 1.2- and 2.5-fold, respectively, from the oral phase to the intestinal phase, and achieved high bioaccessibility (1.2 and 3.1, respectively). *N*-(3-(3,4-dihydroxycinnamoyl)-L-3,4-DOPA (*cis* isomer) was released in the course of the intestinal phase, though *N*-(3-(4-hydroxycinnamoyl)-L-tyrosine was not found in the non-digested CSF but liberated during in vitro digestion. Concerning flavan-3-ols, 2,3,4′,5,7-pentahydroxyflavan-3,4-diol (catechin) increased by 4.1-fold from the oral to the colonic phase. In contrast, 2,3′,4,5′,7-pentahydroxyflavan-3,4-diol (epicatechin) was detected in all stages but the colonic phase, reaching a high bioaccessibility index (1.7). The flavonols (quercetin 3-*O*-glucoside and quercetin 3-*O*-arabinoside) were not detected in the intestinal phase despite being released (33.3%) during the gastric phase. Methylxanthines, including theobromine (525.8 mg 100 g^−1^) and caffeine (169.4 mg 100 g^−1^), which were fully released during digestion, are, in general, almost entirely absorbed in the gastrointestinal tract [34].

The non-digested CSE was also constituted of hydroxybenzoic acids (108.9 mg 100 g^−1^), *N*-phenylpropenoyl-L-amino acids (35.4 mg 100 g^−1^), flavan-3-ols (49.5 mg 100 g^−1^), flavonols (2.7 mg 100 g^−1^), flavones (2.9 mg 100 g^−1^), and a significant quantity of methylxanthines (2639.3 mg 100 g^−1^). Among the hydroxybenzoic acids from the CSE, gallic acid was the main compound (37.6% respect to total phenolics), followed by protocatechuic acid (17.8% respect to total phenolics). Both compounds were released during the digestive process, and notably, protocatechuic acid reached a bioaccessibility index of 1.0. Regarding the *N*-phenylpropenoyl-L-amino acids, *N*-(3-(3,4-dihydroxycinnamoyl)-L-aspartic acid was the most abundant (19.1 mg 100 g^−1^). *N*-(3-(4-Hydroxycinnamoyl)-L-aspartic acid (*trans* isomer) and *N*-(3-(3,4-dihydroxycinnamoyl)-L-DOPA (*cis* isomer) were fully released in the gastric phase and remained stable throughout the course of digestion. Other amino-derived compounds were subsequently degraded throughout the digestion process. Flavan-3-ols, being highly liberated during digestion, increased their concentration from the oral to the colonic phase by 1.7-fold for catechin and 4.6-fold for epicatechin, which achieved a high bioaccessibility value (1.0). In contrast, flavonols were only detected in significant quantities until the gastric phase. The 5,7,4′-Trihydroxyflavone-6,8-di-C-glucoside (apigenin-6,8-di-C-glucoside), which was utterly released in the oral phase, experienced a slight degradation (32.1%) during the intestinal phase and was not detected during the colonic phase. Methylxanthines were again found in large quantities. Theobromine and caffeine showed high bioaccessibility; however, they exhibited a slight degradation (14.8 and 11.7%, respectively) from the oral phase to the colonic phase, in contrast to the sequential release observed in the CSF. Notably, the release of phenolic compounds and methylxanthines during digestion is a multifaceted process influenced by various factors. These factors include the food matrix, the specific digestive enzymes present, and the duration of digestion [35]. Understanding these factors’ interplay is crucial to fully appreciating the digestive process and its implications on the absorption, bioavailability, metabolic fate, and potential biological activity of these compounds.

### 3.2. Digestion Affected Total Phenolic Content and Antioxidant Capacity Depending on the Cocoa Shell Matrix

The TPC of the CSF and the CSE throughout digestion, determined by in vitro analysis, is shown in Table 2. The TPC from the digested phase of the CSF increased by 2.1-fold as digestion proceeded. The TPC in the CSF was associated with the concentration of *N*-coumaroyl-L-tyrosine (*r* = 0.979, *p* < 0.05) (Supplementary Table S1). Meanwhile, the TPC in its non-digested phase decreased by 23.0% from the oral to the colonic phase. The digestion of the CSE caused an increase (1.2-fold) in the TPC of the digested phase. The increase in the TPC of the digested CSF and CSE could be attributed to the release of phenolics from the fibrous matrix. Digestive enzymes can modify the structure of the food matrix, allowing the liberation of phenolic compounds [36].

**Figure 1:**
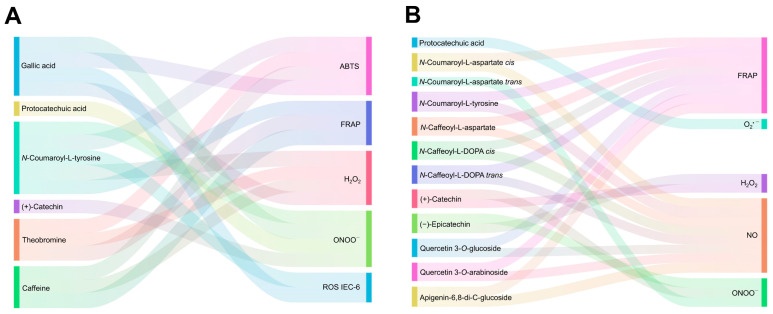
Sankey diagram depicting the significant (*p* < 0.05) Pearson correlations (≥0.8) among the antioxidant properties and the phenolic compounds of the cocoa shell flour (CSF) (**A**) and the cocoa shell extract (CSE) (**B**). Apigenin-6,8-di-C-glucoside: 5,7,4′-Trihydroxyflavone-6,8-di-C-glucoside; Caffeine: 1,3,7-Trimethylxanthine; Catechin: 2,3,4′,5,7-Pentahydroxyflavan-3,4-diol; (−)-Epicatechin: 2,3′,4,5′,7-Pentahydroxyflavan-3,4-diol; Gallic acid: 3,4,5-Trihydroxybenzoic acid; *N*-Caffeoyl-L-aspartate: *N*-(3-(3,4-dihydroxycinnamoyl)-L-aspartic acid; *N*-Caffeoyl-L-DOPA *cis: N*-(3-(3,4-dihydroxycinnamoyl)-L-DOPA; *N*-Caffeoyl-L-DOPA *trans*: *N*-(3-(3,4-dihydroxycinnamoyl)-L-DOPA; *N*-Coumaroyl-L-aspartate *cis: N*-(3-(4-hydroxycinnamoyl)-L-aspartic acid; *N*-Coumaroyl-L-aspartate *trans: N*-(3-(4-hydroxycinnamoyl)-L-aspartic acid; Protocatechuic acid: 3,4-Dihydroxybenzoic acid; Quercetin 3-*O*-arabinoside: 3,3′,4′,5,7-Pentahydroxyflavone 3-β-arabinoside; Quercetin 3-*O*-glucoside: 3,3′,4′,5,7-Pentahydroxyflavone 3-β-glucoside; *N*-Coumaroyl-L-tyrosine: *N*-(3-(4-hydroxycinnamoyl)-L-tyrosine; Theobromine: 3,7-Dimethylxanthine.

The digested CSF showed the highest antioxidant capacity in the colonic phase (38.9 mg TE g^−1^) for the ABTS method and in the intestinal phase (26.8 mg TE g^−1^) for the FRAP method. Nevertheless, an increase in antioxidant activity of 94.9% (ABTS) and 91.1% (FRAP) from the oral to the colonic phases was observed. Concomitantly, the antioxidant capacity in the non-digested residue of CSF decreased by 16.7% for the ABTS method and 22.2% for the FRAP method throughout the digestion. The antioxidant capacity measured by ABTS was associated with the content of gallic acid (*r* = 0.963, *p* < 0.05) and *N*-coumaroyl-L-tyrosine (*r* = 0.984, *p* < 0.01). On the other hand, the antioxidant capacity measured by FRAP was found to be positively correlated with the levels of theobromine and caffeine (*r* = 0.955 and 0.979, *p* < 0.05, respectively). (Figure 1A, Supplementary Table S1). The antioxidant capacity in the CSE measured by the ABTS method experienced an exacerbated increase (6.8-fold). Prior research has demonstrated that the conversion of phenolic compounds throughout the digestive process may result in the formation of novel metabolites possessing improved bioactivity [35]. Consequently, the emergence of these newly formed phenolic metabolites, derived from the transformations produced during digestion, might serve as a plausible reason for the heightened ABTS antioxidant potential detected during the digestion of the CSE [37]. In contrast, when the antioxidant capacity was assessed by the FRAP method, a decrease (46.4%) was observed during digestion, correlating with *N*-coumaroyl-L-tyrosine (*r* = 0.923), *N*-caffeoyl-L-aspartate (*r* = 0.925), *N*-caffeoyl-L-DOPA *trans* (*r* = 0.919), quercetin 3-*O*-glucoside (*r* = 0.918), quercetin 3-*O*-arabinoside (*r* = 0.910), and apigenin-6,8-di-C-glucoside (*r* = 0.932) (*p* < 0.05) (Figure 1B, Supplementary Table S1).

The TPC and the antioxidant capacity quantified spectrophotometrically increased in the digested CSF fractions as digestion occurred. Total phenolics and methylxanthines from the CSF measured individually also increased during digestion. Thus, the liberation of phenolic acids and flavonoids from the non-digested insoluble fiber residue may account for the higher TPC.

On the other hand, in CSE, the TPC and the antioxidant capacity increased along the digestion when they were determined by the ABTS method, and conversely, they decreased when they were analyzed by the FRAP method. Spectrophotometric techniques may occasionally overestimate results due to interactions between reagents, such as Folin–Ciocalteu, and other compounds released during simulated digestion. Additionally, it is crucial to acknowledge that the correlation between phenolic compounds and antioxidant capacity results might be affected by factors such as the presence of non-phenolic antioxidant compounds (e.g., melanoidins, proteins/peptides, and their complexes with phenolic compounds) interacting with spectrophotometric techniques [38]. Furthermore, the structural transformations that phenolic compounds undergo during simulated digestion could enhance their antioxidant capacity [39,40]. Despite these limitations, spectrophotometric methods remain valuable for estimating antioxidant capacity, as phenolic compounds exhibit high antioxidant potential [39]. In the case of CSF and CSE, the increased antioxidant capacity observed using different assessment methods (ABTS and FRAP) implies that various phenolic compounds may display distinct antioxidant potentials depending on the digestion phase [41]. The discrepancies between the ABTS and FRAP methods in quantifying antioxidant capacity underscore the importance of employing multiple techniques to gain a comprehensive understanding of these compounds’ antioxidant potential [42]. Thus, we also assessed the in vitro radical scavenging capacity of the digested CS, targeting physiological radicals (O_2_^•−^, H_2_O_2_, NO^•^, and ONOO^−^).

### 3.3. Phenolic Compounds from the Cocoa Shell May Retain Their Free Radicals Scavenge after Digestion

**Figure 2:**
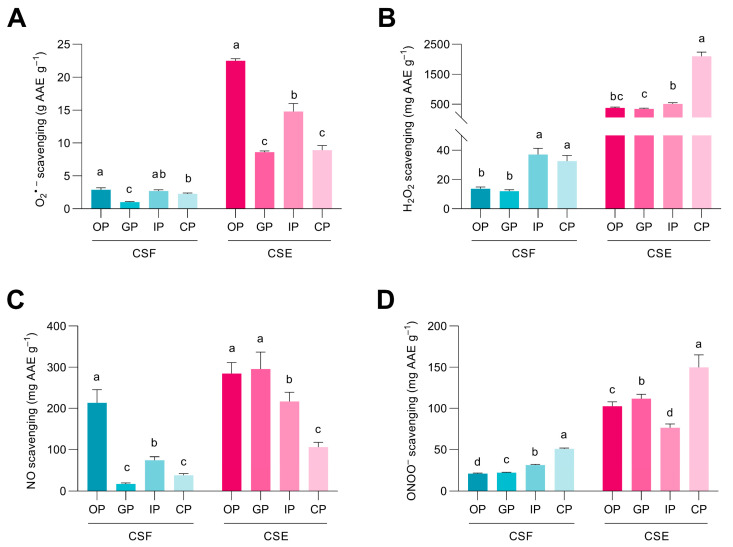
Radical scavenging capacity of the digested fractions from the cocoa shell flour (CSF) and the cocoa shell extract (CSE) for superoxide anion (O_2_^•−^) (**A**), hydrogen peroxide (H_2_O_2_) (**B**), nitric oxide (NO^•^) (**C**), and peroxynitrite (ONOO^−^) (**D**). OP—oral phase; GP—gastric phase; IP—intestinal phase; CP—colonic phase. Bars with different letters denote significant differences between the digested phases of the CSF or the CSE according to ANOVA and Tukey’s multiple range test (*p* < 0.05).

O_2_^•−^ is generated through the reaction between molecular oxygen (O_2_) and electrons during the mitochondrial respiratory chain. However, excessive O_2_^•−^ can be dangerous for the organism, as it is potentially cytotoxic and can interact with other molecules to form highly toxic substances [43]. Phenolic compounds, and especially flavonoids, can scavenge the superoxide radical due to their chemical structure. They can neutralize the superoxide by transferring protons and hydrogen atoms [44]. The O_2_^•−^ scavenging capacity of the digested CSF and CSE fractions is shown in Figure 2A. The CSF’s gastric phase exhibited the lowest O_2_^•−^ scavenging capacity, increasing by 2.7-fold in the course of the intestinal phase and by 2.3-fold at the end of the colonic phase, presumably because of the release of phenolic compounds with O_2_^•−^ radical scavenging activity. In contrast, the CSE’s oral phase showed the highest O_2_^•−^ radical scavenging, decreasing by 60.4% from the oral to the colonic phase, probably due to the degradation and transformation of phenolic compounds into smaller phenols with lower O_2_^•−^ radical scavenging capacity. The O_2_^•−^ scavenging activity significantly correlated with the content of protocatechuic acid in CSE (*r* = 0.969, *p* < 0.05) (Figure 1B; Supplementary Table S1).

O_2_^•−^ can be neutralized by mitochondrial and cytosolic SODs. However, its conversion generates H_2_O_2_, which can also engender toxicity, principally when transformed into other ROS such as ^•^OH (by the Fenton reaction). The ability of phenolic compounds to remove H_2_O_2_ lies in their hydrogen-donating capacity [45]. The intestinal and colonic bioaccessible fractions of CSF hold the strongest scavenging ability of H_2_O_2_ (37.0 and 32.5 mg AAE g^−1^, respectively) (Figure 2B). The digestive process increased the H_2_O_2_ trapping capacity 2.4-fold from the oral to the colonic phases in CSF. The H_2_O_2_ scavenging capacity showed a similar behavior in CSE, displaying an exacerbated increase (5.7-fold) throughout digestion. Particularly, *N*-coumaroyl-L-tyrosine correlated significantly with the H_2_O_2_ scavenging capacity (*r* = 0.988, *p* < 0.01) in CSF and epicatechin (*r* = 0.947, *p* < 0.01) in CSE (Figure 1A,B; Supplementary Table S1).

NO is produced in biological tissues by nitric oxide synthases through the reaction of H_2_O_2_ with arginine. NO has limited chemical reactivity and low direct toxicity, intervening in many physiological functions. Nonetheless, when NO reacts with other radicals, it can produce highly toxic molecules, leading to cellular damage and impaired function [46]. Gastrointestinal digestion reduced the NO scavenging capacity of the CSF and the CSE (Figure 2C). The oral phase of the CSF reached a NO trapping capacity of 213.1 mg AAE g^−1^, which decreased by 82.2% by the end of the colonic phase. Similarly, the CSE’s NO scavenging capacity was high in the first stages of digestion, reaching 284.5 mg AAE g^−1^ during the oral phase, but decreased by 62.8% from the oral to the colonic phase. The NO scavenging capacity was significantly associated with the content of *N*-Coumaroyl-L-tyrosine (*r* = 0.982, *p* < 0.01), *N*-Caffeoyl-L-DOPA *cis* (*r* = 0.921, *p* < 0.05), and apigenin-6,8-di-C-glucoside (*r* = 0.993, *p* < 0.01) in the CSE (Figure 1B; Supplementary Table S1).

ONOO^•−^ is a highly toxic molecule produced by the reaction between the radicals O_2_^•−^ and NO^•^. ONOO^•−^ can diffuse through cell membranes, causing damage to lipids or DNA [46]. Phenolic compounds are able to scavenge ONOO^•−^ through electron donation; thus, the radical scavenging capacity increases with the number of hydroxyl groups in the phenolic structures [47]. The ONOO^−^ scavenging capacity in CSF increased 2.4-fold from the oral phase to the colonic phase, reaching 50.6 mg AAE g^−1^ in the colonic phase (Figure 2D). Meanwhile, the CSE‘s ONOO^−^ scavenging capacity decreased by 25.6% from the oral to the intestinal phase. During the colonic phase the ONOO^−^ scavenging capacity was recovered, achieving the highest value (149.6 mg AAE g^−1^) compared to the rest of the CSE’s digested bioaccessible fractions. The ONOO^−^ scavenging capacity in the CSF was mainly associated with the content of gallic (*r* = 0.985, *p* < 0.01) and protocatechuic acids (*r* = 0.925, *p* < 0.05) (Figure 1A, Supplementary Table S1). Meanwhile, in CSE, it was associated with the concentration of catechin and epicatechin (*r* = 0.940 and 0.922, *p* < 0.05, respectively) (Figure 1B, Supplementary Table S1). The consistently higher scavenging capacity in the CSE compared to the CSF highlights the food matrix’s importance in influencing these compounds’ antioxidant potential during digestion. This observation can be attributed to the increased concentration of phenolic compounds in the digested CSE, which enhances their availability to interact with the reactive species under investigation [48]. The scavenging capacity is particularly remarkable for H_2_O_2_, followed by NO^•^ and ONOO^−^, with O_2_^•−^ exhibiting the lowest capacity. The observed changes in the radical scavenging capacities of the digested CS fractions can be attributed to the dynamic release, transformation, and degradation of phenolic compounds during digestion [49]. Previous studies have reported that the antioxidant potential of phenolic compounds is highly dependent on their chemical structure and concentration [50].

### 3.4. In Vitro Simulated Digestion Maintained the Cocoa Shell’s Capacity to Decrease ROS Production in Intestinal and Hepatic Cells

**Figure 3:**
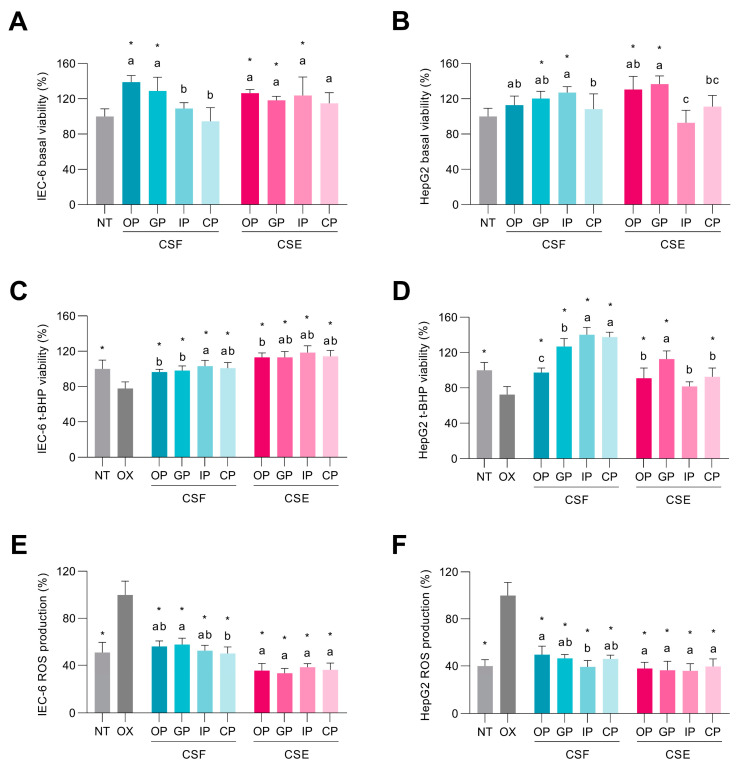
Effect of the digested fractions from the cocoa shell flour (CSF) and the cocoa shell extract (CSE) on basal cell viability in intestinal (**A**) and hepatic cells (**B**). Protective effect of the digested CSF and CSE against *tert*-butyl hydroperoxide (*t*-BHP, 1 mmol L^−1^) cell viability reduction in intestinal (**C**) and hepatic cells (**D**) and ROS production in intestinal (**E**) and hepatic cells (**F**). Bars with different letters denote significant differences between the digested phases of the CSF or the CSE according to ANOVA and Tukey’s multiple range test (*p* < 0.05). NT—non-treated control cells; OP—oral phase; GP—gastric phase; IP—intestinal phase; CP—colonic phase. Asterisks (*) indicate differences between samples and the oxidized cells (OX) group according to Dunnett’s test (*p* < 0.05).

The basal viability determined in intestinal IEC-6 cells treated with the different digested fractions of CSF and CSE is shown in Figure 3A. Intestinal cells treated with the oral and gastric fractions of the CSF increased their viability by 38.8% and 28.8%, respectively, compared to non-treated cells. Similarly, when IEC-6 cells were treated with oral, gastric, and intestinal fractions of the CSE, their viability was also significantly increased. HepG2 cells treated with the different digested fractions from the CSF showed higher basal viability when they were treated with the gastric (120.3%) and intestinal (127.2%) digestion fractions, compared to non-treated cells (Figure 3B). The oral and gastric fractions of CSE were noted for increasing hepatocyte viability by 30.5% and 36.7%, respectively, compared to non-treated cells.

The viability of intestinal cells challenged with *t*-BHP decreased by 22.2% compared to non-treated cells (Figure 3C). The bioaccessible fractions of both the CSF and the CSE protected cell viability (96.5–118.6%) compared to the cells treated solely with *t*-BHP (77.8%). As shown in Figure 1A and Supplementary Table S1, the viability of the intestinal cells correlated significantly with the concentrations of *N*-coumaroyl-L-tyrosine and caffeine (*r* = 0.915 and 0.905, *p* < 0.05, respectively) in the CSF. Hepatocytes treated with *t*-BHP showed the same behavior, reducing their viability by 27.6% with respect to non-treated cells (Figure 3D). The digested CSF evoked a significantly higher cell viability (97.4–140.3%) compared to cells treated only with *t*-BHP (72.4%). Viability in the hepatic cells was significantly associated with the content of theobromine (*r* = 0.971, *p* < 0.05) and caffeine (*r* = 0.989, *p* < 0.01) in the CSF (Figure 1A; Supplementary Table S1). On the other hand, hepatocytes also presented higher viability (91.0–112.6%) when co-treated with the digested fractions of the CSE, excluding the intestinal fraction. The cytoprotective effect of the digested bioaccessible fractions of the CSF and the CSE can be attributed to their rich phenolic compound concentrations. These compounds have demonstrated potential for safeguarding cells against oxidative stress-induced cytotoxicity by modulating cellular antioxidant signaling pathways, thereby promoting cell survival and averting cell death [51].

The *t*-BHP treatment induced oxidative stress in intestinal cells, increasing ROS production by 49.0% compared to non-treated cells (Figure 3E). However, all the digested CSF and CSE prevented oxidative stress. The bioaccessible fraction from the CSF and the CSE significantly blocked ROS production increases compared to *t*-BHP-elicited intestinal cells. ROS scavenging in the intestinal cells correlated (*p* < 0.05) with the concentration of gallic acid (*r* = 0.965), and *N*-coumaroyl-L-tyrosine (*r* = 0.930) in the CSF (Figure 1A; Supplementary Table S1). Hepatic *t*-BHP-stimulated cells suffered exacerbated oxidative stress compared to non-treated cells, as the ROS level was 2.5-fold higher compared to non-treated cells (Figure 3F). All the digested CSF and CSE fractions scavenged ROS, yielding significantly lower ROS levels. Our findings indicate that pre-treatment for 24 h and co-treatment for 1 h with all CSF and CSE digestion fractions can effectively prevent oxidative stress by inhibiting ROS generation, primarily due to their abundant antioxidant compounds. Phenolic compounds, particularly hydroxybenzoic acids, have been reported to possess the ability to reduce ROS production in intestinal and hepatic cells, thereby contributing to the protective effects of CSF and CSE during digestion [51]. Specifically, protection against ROS correlated significantly with the total concentration of *N*-phenylpropenoyl-L-amino acids (*r* = 0.903, *p* < 0.05) in CSF (Figure 1A; Supplementary Table S1). In the literature, various studies have demonstrated the potential of phenolic compounds to modulate cellular redox status and protect cells against oxidative damage by scavenging free radicals and chelating metal ions [46]. These compounds can also influence the activity of antioxidant enzymes and regulate signaling pathways involved in the cellular response to oxidative stress [52]. As a result, the presence of phenolic compounds in digested CSF and CSE fractions is likely a key factor contributing to their observed antioxidant and cytoprotective effects.

### 3.5. The Digested Cocoa Shell Activated the Cellular Antioxidant Defense System in Intestinal and Hepatic Cells

The organism possesses an antioxidant defense system to counteract the effects of oxidants composed of enzymatic and non-enzymatic antioxidants. The main enzymatic antioxidants are SOD, CAT, and GPx. Meanwhile, non-enzymatic antioxidants include vitamins, β-carotene, uric acid, and GSH, a tripeptide comprising a thiol group [53]. In this study, the levels of GSH and thiol groups and the activities of the antioxidant enzymes SOD and CAT were evaluated in intestinal and hepatic cells.

**Figure 4:**
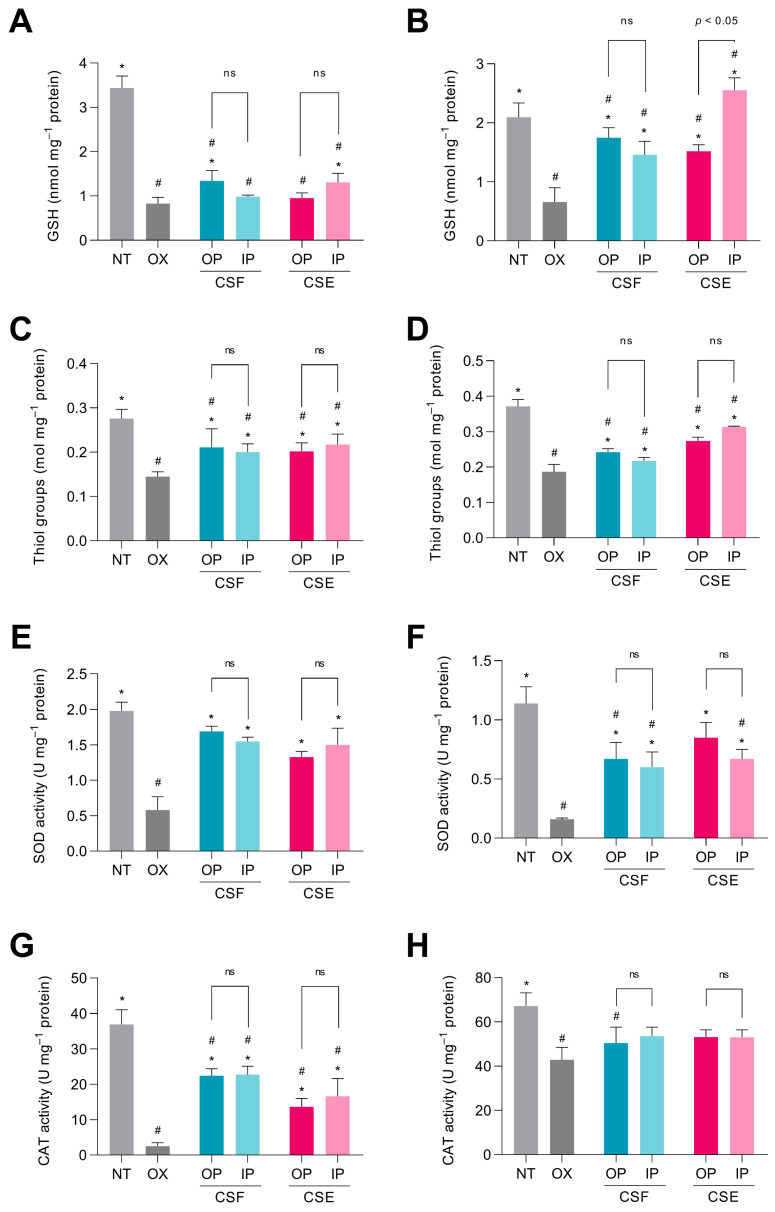
Effect of the digested cocoa shell flour (CSF) and the cocoa shell extract (CSE) (oral and intestinal fractions) on *tert*-butyl hydroperoxide (*t*-BHP, 1 mmol L^−1^)-derived dysregulation of the cellular antioxidant response, including glutathione levels in IEC-6 (**A**) and HepG2 cells (**B**), thiol levels in IEC6 (**C**) and HepG2 cells (**D**), superoxide dismutase (SOD) activity (U mg^−1^) in IEC-6 (**E**) and HepG2 cells (**F**), and catalase (CAT) activity (U mg^−1^) in IEC-6 (**G**) and HepG2 cells (**H**). OP—oral phase; IP—intestinal phase. Asterisks (*) and hashes (^#^) indicate differences between samples and oxidized cells (OX) and the non-treated control (NT) groups, respectively, according to Dunnett’s test (*p* < 0.05); ns: non-significant differences.

GSH is a highly abundant and soluble antioxidant that donates its electron to H_2_O_2_, promoting its reduction to H_2_O and O_2_ [53]. GSH levels decreased 76.1% in intestinal cells stimulated with *t*-BHP compared with non-treated intestinal cells (Figure 4A). The oral and intestinal fractions from the CSF and the CSE, respectively, sustained significantly higher GSH levels than cells only treated with the oxidant. Hepatocytes treated with *t*-BHP also showed a lower GSH level (68.4%) than non-treated cells (Figure 4B). It was observed that GSH levels in cells treated with the oral and intestinal fractions from the CSF were 2.6- and 2.2-fold higher, respectively, than in *t*-BHP-stimulated hepatic cells. Similarly, the oral and intestinal fractions of the CSE triggered higher GSH levels (2.3- and 3.9-fold, respectively) than *t*-BHP-treated hepatic cells.

*t*-BHP can be detoxified by cytosolic glutathione peroxidases via reduction to *tert*-butyl alcohol, leading to the depletion of GSH levels by conversion to glutathione disulfide (GSSG) [54]. Our results indicate that co-treatment with the digested fractions of the CSF and the CSE, which are rich in phenolic compounds, can potentially increase intracellular GSH levels, thereby strengthening cellular defense against oxidative stress. In the literature, it has been well documented that phenolic compounds play an important role in the cellular antioxidant defense system by modulating the intracellular levels of GSH [55]. Phenolic compounds may also influence the expression of genes involved in GSH synthesis and regeneration (they are involved in GSH synthesis by increasing the expression of the enzyme γ-glutamylcysteine synthetase) [56]. This highlights the potential of CSF and CSE as sources of bioactive compounds that can be utilized for their antioxidant and cytoprotective properties.

Excessive oxidative stress causes oxidation of thiols in mainly peptides and proteins, leading to alteration of protein structure and function [57]. As shown in Figure 4C, the thiol concentration in the non-treated intestinal cells was 1.9-fold higher than in the *t*-BHP-treated cells. IEC-6 cells co-treated with *t*-BHP and with the digested fractions of the CSF and the CSE showed a significantly higher thiol concentration than cells treated with only the oxidant. *t*-BHP induced a decrease of 49.6% in thiol levels in hepatic cells (Figure 4D). The oral fraction of the CSF preserved a 22.7% higher thiol concentration than cells treated only with *t*-BHP. The same behavior was observed in hepatocytes treated with the oral and intestinal digestive fractions of the CSE, which showed 1.5 and 1.7-fold higher thiol concentrations, respectively, when compared with *t*-BHP-stimulated hepatocytes. Cells treated with *t*-BHP exhibited lower concentrations of thiol groups, probably by reaction with reactive oxygen, nitrogen, or sulfur species [58]. Interestingly, cells were protected against thiol group degradation when treated with the digested fractions of both CS matrices. This protection can be attributed to the antioxidant properties of phenolic compounds present in the digested CSF and CSE fractions, which can help mitigate oxidative stress by neutralizing reactive species and preventing thiol oxidation [55].

SOD serves as the first line of defense against oxidative stress generated by ROS, removing O_2_^•−^, which could produce cellular damage, by catalyzing its dismutation into molecular oxygen and H_2_O_2_ [59]. In Figure 4E, it was observed that *t*-BHP stimulation caused a reduction of 70.7% in the SOD enzyme activity in IEC-6 cells compared to non-treated cells. However, co-treatment of the cells with *t*-BHP and either the digestive fractions of the CSF or the CSE prevented the decline in SOD activity. No significant differences were observed between the digestive fractions of CSF and CSE. The results in Figure 4F showed that *t*-BHP stimulation caused a decrease of 86.0% in SOD activity in hepatic cells compared to the basal level. Nevertheless, SOD activity in cells co-treated with the oxidant and the CS was up to 5.3-fold higher than in cells treated only with *t*-BHP, indicating the protective effect of phenolic compounds against oxidative stress. The H_2_O_2_ produced by the dismutation of O_2_^•−^ through the action of SOD can further generate ^•^OH in the presence of Fe^2+^, leading to oxidative damage. Alternatively, H_2_O_2_ can be degraded into molecular oxygen and H_2_O by the activity of CAT [60]. CAT activity was markedly decreased by 93.2% in intestinal cells stimulated with *t*-BHP (Figure 4G). Although the digested fractions of the CSF and the CSE did not fully prevent CAT activity loss, it is important to note that they preserved higher CAT activity (32.5–58.8%) than cells treated only with *t*-BHP. CAT activity in *t*-BHP-stimulated hepatocytes was significantly (*p* < 0.05) low (36.2%) compared to non-treated cells (Figure 4H). In general, hepatic cells co-treated with *t*-BHP and the digested fractions of the CSF and the CSE did not recover their CAT activity significantly. The observed protective effects of CSF and CSE could be attributed to their phenolic compounds, which help maintain SOD and CAT activity in response to oxidative stress, thereby promoting cellular health [55]. The protective effects of CSF and CSE on SOD and CAT activity could be linked to their potential role in modulating the nuclear factor erythroid 2-related factor 2 (Nrf2)/antioxidant response element (ARE) signaling pathway. The Nrf2/ARE signaling pathway plays a crucial role in the cellular response to oxidative stress. Under normal conditions, Nrf2 is bound to Kelch-like ECH-associated protein 1 (Keap1) in the cytoplasm [52]. Upon exposure to oxidative stress or electrophilic compounds, Nrf2 is released from Keap1 and translocates to the nucleus, where it binds to ARE in the promoter regions of target genes. This binding event leads to the expression of antioxidant and detoxification enzymes, including SOD and CAT, which help to neutralize the detrimental effects of ROS and restore cellular homeostasis [7]. Phenolic compounds present in CSF and CSE may act as activators of the Nrf2/ARE pathway. Phenolic compounds possess electrophilic properties, allowing them to modify cysteine residues in Keap1, thereby facilitating Nrf2 release and subsequent nuclear translocation, resulting in enhanced expression of antioxidant enzymes and increased cellular resistance to oxidative stress [52].

### 3.6. The Antioxidant and Radical Scavenging Capacities of the Cocoa Shell Are Influence by the Phytochemical Composition, Digestion, and Matrix

**Figure 5:**
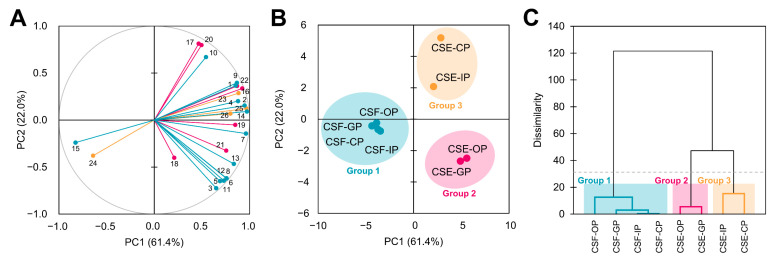
Principal component analysis (**A**,**B**). Agglomerative hierarchical cluster analysis (**C**) illustrating the behavior of phenolic compounds and methylxanthines from the cocoa shell during simulated gastrointestinal digestion. Number identification: 1: Gallic acid; 2: Protocatechuic acid; 3: *N*-Coumaroyl-L-aspartate *cis*; 4: *N*-Coumaroyl-L-aspartate *trans*; 5: *N*-Coumaroyl-L-tyrosine; 6: *N*-Caffeoyl-L-aspartate; 7: *N*-Caffeoyl-L-DOPA *cis*; 8: *N*-Caffeoyl-L-DOPA *trans*; 9: (+)-Catechin; 10: (−)-Epicatechin; 11: Quercetin 3-*O*-glucoside; 12: Quercetin 3-*O*-arabinoside; 13: Apigenin-6,8-di-C-glucoside; 14: Theobromine; 15: Caffeine; 16: TPC (Total Phenolic Content); 17: ABTS antioxidant capacity; 18: FRAP antioxidant capacity; 19: O_2_^•−^ scavenging; 20: H_2_O_2_ scavenging; 21: NO scavenging; 22: ONOO^−^ scavenging; 23: viability in IEC-6 cells; 24: viability in HepG2 cells; 25: ROS scavenging in IEC-6 cells; 26: ROS scavenging in HepG2 cells. OP—oral phase; GP—gastric phase; IP—intestinal phase; CP—colonic phase.

For further investigating the relationship of the CS phytochemical composition with the antioxidant properties of the CSF and the CSE through gastrointestinal digestion, we computed multivariate analyses (PCA and hierarchical cluster analysis) (Figure 5). Five different principal components (PCs) were obtained. Figure 5A depicts the PC loadings for the first two PCs. We can observe that PC1 explained 61.4% of the whole variability and integrated variables such as ROS scavenging in IEC-6 cells (5.9%), the concentration of theobromine (5.9%), *N*-caffeoyl-L-DOPA *cis* concentration (5.8%), protocatechuic acid concentration (5.6%), and TPC (5.3%). Conversely, the PC2 (explaining 22.0% of the variability) comprised variables such as ABTS (11.6%), H_2_O_2_ scavenging (11.1%), and the concentration of *N*-coumaroyl-L-aspartate *cis* (9.2%) and (−)-epicatechin (7.9%).

Consequently, PC scores (Figure 5B) and hierarchical cluster analysis (Figure 5C) classified samples into three distinct groups. Group 1 encompassed all CSF digestion fractions and was characterized by low TPC, radical, and ROS scavenging but a high caffeine concentration. Group 2 consisted of the oral and gastric fractions of the CSE. These samples were characterized by higher FRAP antioxidant capacity and NO scavenging, as well as increased concentrations of some *N*-phenylpropenoyl-L-amino acids and flavonoids (both flavonols and flavones). Finally, group 3 included the two remaining samples, the intestinal and colonic bioaccessible fractions of the CSE, and was characterized by high ABTS antioxidant capacity, H_2_O_2_ scavenging, and (−)-epicatechin concentration. Those results proved that the CSF phenolic composition was less altered by the course of gastrointestinal digestion, whereas the CSE composition, and therefore its antioxidant and radical scavenging properties, were highly affected by digestion. Then, digestion and matrix (flour vs. extract) influenced the CS phytochemical composition and antioxidant capacity.

**Figure 6:**
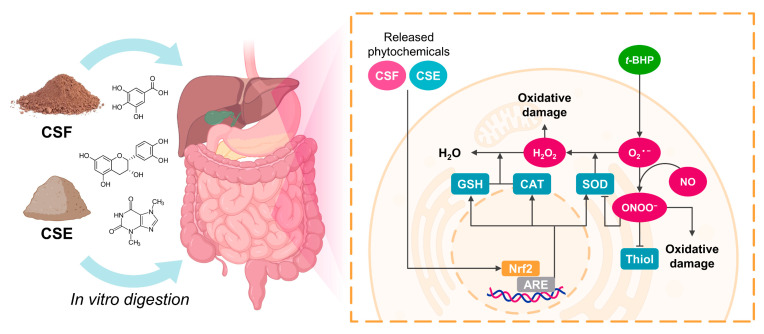
An illustrated diagram integrating the effects of the phytochemicals released from the CSF and the CSE during the simulated gastrointestinal digestion on the prevention of oxidative stress in intestinal and hepatic cells.

Figure 6 summarizes the effects of the phytochemicals released from the CSF and the CSE on the course of gastrointestinal digestion and the prevention of oxidative stress in intestinal and hepatic cells. Upon exposure to *t*-BHP, cell mitochondria may sustain damage, leading to increased production of mitochondrial ROS, specifically O_2_^•−^. The CS’ phenolic compounds, such as other phytochemicals, can elicit the Nrf2/ARE signaling pathway and subsequently enhance the activity of downstream antioxidant enzymes such as SOD, CAT, or the expression of GSH [52]. Thus, the CS phenolic compounds may promote SOD-mediated conversion of O_2_^•−^ into H_2_O_2_, which can then be decomposed into H_2_O by the action of CAT and GSH. Low concentrations of H_2_O_2_ can induce oxidative damage, either directly or by transforming into ^•^OH. This process may trigger the activation of inflammation-associated transcription factors, such as nuclear factor kappa B (NF-κB) and activator protein 1 (AP-1), potentially resulting in inflammatory intestinal or liver diseases [61]. Concurrently, NO might rapidly react with O_2_^•−^ occurs ONOO^−^. It can react with thiols, altering the function of proteins or disrupting cellular signaling pathways, and inactivate SOD by oxidizing its active site or by nitrosylating its cysteine residues, leading to a decrease in SOD activity and an increase in superoxide radicals [62]. Owing to its high reactivity, ONOO^−^ can cause cellular damage and induce apoptosis (programmed cell death) or necrosis (cell death due to injury or disease) by causing oxidative stress and activating cell death signaling pathways [62].

The antioxidant and radical scavenging properties of the CS are significantly influenced by its phytochemical composition, gastrointestinal digestion, and food matrix. This study demonstrated that the gastrointestinal digestion process and the food matrix played a crucial role in determining the phytochemical composition and, thereafter, the associated antioxidant and radical scavenging capacities of the CS. Phenolic compounds present in this by-product may protect intestinal and hepatic cells against oxidative stress by activating the Nrf2/ARE signaling pathway, enhancing the activity of antioxidant enzymes such as SOD and CAT, and increasing GSH and thiol levels. Consequently, the CS’s phenolic compounds may prevent inflammation and cellular damage by neutralizing harmful ROS and RNS. This cocoa by-product could potentially be a valuable source of natural antioxidants and radical scavengers, which could be harnessed for their health-promoting properties in the prevention and treatment of oxidative stress-related diseases. The CSF and the CSE could be effectively incorporated into various novel products in different food categories, including nutritional supplements and gluten-free formulations, to meet the increasing demand for functional and health-enhancing foods. Upcoming research and product development will help optimize processing methods and maximize the beneficial effects of CS-derived phytochemicals in a diverse range of food applications. Further research is necessary to fully understand the bioavailability and bioactivity of these compounds in vivo as well as their potential applications in functional foods, nutraceuticals, and pharmaceuticals.

## 4. Conclusions

In this study, we investigated for the first time the impact of simulated gastrointestinal digestion on the concentration of phenolic compounds in the CSF and the CSE and their consequent radical scavenging capacity and antioxidant activity in intestinal epithelial and hepatic cells. Our results showed that both CSF and CSE are rich in methylxanthines and phenolic compounds, including theobromine, caffeine, gallic acid, and (+)-catechin, which remained present throughout the digestion process. Importantly, gastrointestinal digestion enhanced the in vitro antioxidant capacity of CSF and CSE and increased their free radical scavenging capacity. Additionally, CSF and CSE, not exhibiting cytotoxicity in IEC-6 and HepG2 cells, effectively counteracted *t*-BHP-induced oxidative stress and preserved GSH and thiol levels and SOD and CAT activities in both cell types. These findings highlight the potential of the CS phenolic compounds as functional food ingredients that can promote health and combat cellular oxidative stress associated with chronic disease development. Further research could help optimize the processing methods to maximize the beneficial effects of CS’s phytochemicals in functional foods and nutraceuticals.

## Figures and Tables

**Table 1:** Concentration of individual phenolic compounds and methylxanthines (mg 100 g^−1^) in the non-digested and digested cocoa shell flour (CSF) and extract (CSE) throughout the different phases of the simulated gastrointestinal digestion.

Compounds	ND	OP	GP	IP ^†^		CP
Cocoa shell flour						
*Hydroxybenzoic acids*						
3,4,5-Trihydroxybenzoic acid (Gallic acid)	16.0 ± 0.4 ^a^	7.2 ± 0.6 ^c^	7.1 ± 0.4 ^c^	11.8 ± 0.8 ^b^	(0.7)	16.5 ± 1.5 ^a^
3,4-Dihydroxybenzoic acid (Protocatechuic acid)	6.6 ± 0.6 ^b^	2.7 ± 0.1 ^d^	5.4 ± 0.0 ^bc^	4.6 ± 0.3 ^c^	(0.7)	10.8 ± 1.0 ^a^
*Total*	22.6 ± 1.0 ^b^	9.9 ± 0.6 ^d^	12.5 ± 0.4 ^d^	16.5 ± 1.0 ^c^	(0.7)	27.3 ± 2.5 ^a^
*N-Phenylpropenoyl-L-amino acids*						
*N*-(3-(4-Hydroxycinnamoyl)-L-aspartic acid (*N*-Coumaroyl-L-aspartate) *cis*	0.9 ± 0.0 ^b^	0.9 ± 0.0 ^b^	0.8 ± 0.1 ^b^	1.1 ± 0.1 ^a^	(1.2)	0.8 ± 0.1 ^b^
*N*-(3-(4-Hydroxycinnamoyl)-L-aspartic acid (*N*-Coumaroyl-L-aspartate) *trans*	0.5 ± 0.0 ^b^	0.6 ± 0.0 ^b^	0.5 ± 0.1 ^b^	1.5 ± 0.1 ^a^	(3.1)	n.d
*N*-(3-(4-Hydroxycinnamoyl)-L-tyrosine (*N*-Coumaroyl-L-tyrosine)	n.d.	0.1 ± 0.0 ^b^	0.1 ± 0.0 ^b^	0.4 ± 0.0 ^a^	n.d.	0.4 ± 0.0 ^a^
*N*-(3-(3,4-Dihydroxycinnamoyl)-L-aspartic acid (*N*-Caffeoyl-L-aspartate)	5.1 ± 0.3	n.d.	n.d.	n.d.	(0.0)	n.d.
*N*-(3-(3,4-Dihydroxycinnamoyl)-L-3,4- DOPA (*N*-Caffeoyl-L-DOPA) *cis*	0.9 ± 0.0 ^a^	0.6 ± 0.0 ^c^	0.6 ± 0.0 ^bc^	0.4 ± 0.1 ^d^	(0.5)	0.7 ± 0.1 ^b^
*Total*	7.4 ± 0.4 ^a^	2.1 ± 0.1 ^c^	2.0 ± 0.1 ^c^	3.4 ± 0.3 ^b^	(0.5)	2.0 ± 0.2 ^c^
*Flavan-3-ols*						
2,3,4′,5,7-Pentahydroxyflavan-3,4-diol ((+)-Catechin)	11.5 ± 0.8 ^b^	4.0 ± 0.4 ^d^	10.0 ± 0.2 ^b^	7.2 ± 0.6 ^c^	(0.6)	16.5 ± 1.4 ^a^
2,3′,4,5′,7-Pentahydroxyflavan-3,4-diol ((−)-Epicatechin)	1.3 ± 0.1 ^b^	1.5 ± 0.1 ^b^	2.0 ± 0.1 ^a^	2.3 ± 0.1 ^a^	(1.7)	n.d.
*Total*	12.8 ± 0.9 ^b^	5.5 ± 0.5 ^d^	12.0 ± 0.3 ^b^	9.4 ± 0.7 ^c^	(0.7)	16.5 ± 1.4 ^a^
*Flavonols*						
3,3′,4′,5,7-Pentahydroxyflavone 3-β-glucoside (Quercetin 3-*O*-glucoside)	0.3 ± 0.0 ^a^	0.1 ± 0.0 ^b^	0.1 ± 0.0 ^b^	n.d.	(0.0)	n.d.
3,3′,4′,5,7-Pentahydroxyflavone 3-β-arabinoside (Quercetin 3-*O*-arabinoside)	0.3 ± 0.0 ^a^	0.1 ± 0.0 ^b^	0.1 ± 0.0 ^c^	n.d.	(0.0)	n.d.
*Total*	0.6 ± 0.0 ^a^	0.2 ± 0.0 ^b^	0.2 ± 0.0 ^b^	n.d.	(0.0)	n.d.
*Methylxanthines*						
3,7-Dimethylxanthine (Theobromine)	525.8 ± 4.9 ^a^	236.0 ± 2.0 ^d^	382.2 ± 41.1 ^c^	470.1 ± 12.8 ^b^	(0.9)	510.6 ± 3.1 ^ab^
1,3,7-Trimethylxanthine (Caffeine)	169.4 ± 0.4 ^a^	55.8 ± 0.1 ^d^	101.0 ± 1.1 ^c^	130.3 ± 1.0 ^b^	(0.8)	132.9 ± 3.1 ^b^
*Total*	695.2 ± 5.2 ^a^	291.8 ± 2.1 ^d^	483.2 ± 42.2 ^c^	600.5 ± 13.8 ^b^	(0.9)	643.5 ± 6.2 ^b^
Cocoa shell extract						
*Hydroxybenzoic acids*						
3,4,5-Trihydroxybenzoic acid (Gallic acid)	73.9 ± 2.2 ^a^	64.1 ± 1.7 ^b^	31.1 ± 2.4 ^d^	39.2 ± 2.6 ^c^	(0.5)	66.7 ± 6.3 ^ab^
3,4-Dihydroxybenzoic acid (Protocatechuic acid)	34.9 ± 2.4 ^b^	40.2 ± 0.7 ^a^	27.7 ± 0.7 ^d^	33.4 ± 2.3 ^bc^	(1.0)	31.0 ± 1.1 ^cd^
*Total*	108.9 ± 4.7 ^a^	104.3 ± 2.4 ^ab^	58.8 ± 3.2 ^d^	72.6 ± 4.9 ^c^	(0.7)	97.7 ± 7.5 ^b^
*N-Phenylpropenoyl-L-amino acids*						
*N*-(3-(4-Hydroxycinnamoyl)-L-aspartic acid (*N*-Coumaroyl-L-aspartate) *cis*	6.2 ± 0.6 ^a^	5.9 ± 0.0 ^a^	6.4 ± 6.0 ^a^	n.d.	(0.0)	n.d.
*N*-(3-(4-Hydroxycinnamoyl)-L-aspartic acid (*N*-Coumaroyl-L-aspartate) *trans*	4.5 ± 0.3 ^bc^	4.9 ± 0.4 ^b^	4.1 ± 0.0 ^c^	1.7 ± 0.0 ^d^	(0.4)	5.6 ± 0.3 ^a^
*N*-(3-(4-Hydroxycinnamoyl)-L-tyrosine (*N*-Coumaroyl-L-tyrosine)	0.8 ± 0.2 ^bc^	1.0 ± 0.0 ^b^	1.3 ± 0.1 ^a^	0.6 ± 0.1 ^c^	(0.8)	n.d.
*N*-(3-(3,4-Dihydroxycinnamoyl)-L-aspartic acid (*N*-Caffeoyl-L-aspartate)	19.1 ± 1.8 ^ab^	21.1 ± 0.7 ^a^	17.3 ± 2.0 ^b^	n.d.	(0.0)	n.d.
*N*-(3-(3,4-Dihydroxycinnamoyl)-L-3,4-DOPA (*N*-Caffeoyl-L-DOPA) *cis*	3.8 ± 0.1 ^b^	3.8 ± 0.4 ^b^	4.7 ± 0.5 ^a^	3.1 ± 0.3 ^c^	(0.8)	2.4 ± 0.0 ^c^
*N*-(3-(3,4-Dihydroxycinnamoyl)-L-3,4-DOPA (*N*-Caffeoyl-L-DOPA) *trans*	1.1 ± 0.0 ^a^	1.1 ± 0.1 ^a^	1.0 ± 0.0 ^a^	n.d.	(0.0)	n.d.
*Total*	35.4 ± 3.0 ^a^	37.8 ± 1.6 ^a^	34.7 ± 3.3 ^a^	5.4 ± 0.4 ^b^	(0.2)	8.0 ± 0.3 ^b^
*Flavan-3-ols*						
2,3,4′,5,7-Pentahydroxyflavan-3,4-diol ((+)-Catechin)	46.1 ± 1.4 ^bc^	41.6 ± 3.8 ^c^	56.1 ± 5.6 ^b^	40.3 ± 0.2 ^c^	(0.9)	71.4 ± 8.3 ^a^
2,3′,4,5′,7-Pentahydroxyflavan-3,4-diol ((−)-Epicatechin)	3.5 ± 0.1 ^c^	3.0 ± 0.4 ^c^	6.0 ± 0.5 ^b^	3.3 ± 0.3 ^c^	(1.0)	13.8 ± 1.5 ^a^
*Total*	49.5 ± 1.5 ^c^	44.6 ± 4.2 ^c^	62.1 ± 6.2 ^b^	43.7 ± 0.6 ^c^	(0.9)	85.1 ± 9.8 ^a^
*Flavonols*						
3,3′,4′,5,7-Pentahydroxyflavone 3-β-glucoside (Quercetin 3-*O*-glucoside)	1.4 ± 0.0 ^a^	1.3 ± 0.0 ^b^	1.2 ± 0.0 ^b^	n.d.	(0.0)	n.d.
3,3′,4′,5,7-Pentahydroxyflavone 3-β-arabinoside (Quercetin 3-*O*-arabinoside)	1.3 ± 0.0 ^a^	1.3 ± 0.1 ^a^	1.3 ± 0.0 ^a^	n.d.	(0.0)	n.d.
*Total*	2.7 ± 0.1 ^a^	2.5 ± 0.2 ^a^	2.5 ± 0.1 ^a^	–	(0.0)	–
*Flavones*						
5,7,4′-Trihydroxyflavone-6,8-di-C-glucoside (Apigenin-6,8-di-C-glucoside)	2.9 ± 0.0 ^b^	2.8 ± 0.2 ^b^	3.4 ± 0.1 ^a^	1.9 ± 0.2 ^c^	(0.7)	n.d.
*Methylxanthines*						
3,7-Dimethylxanthine (Theobromine)	2605.3 ± 125.5 ^a^	2253.0 ± 20.6 ^b^	1759.8 ± 125.9 ^c^	1249.8 ± 49.0 ^d^	(0.5)	1919.3 ± 86.3 ^c^
1,3,7-Trimethylxanthine (Caffeine)	34.0 ± 2.0 ^a^	28.1 ± 0.8 ^b^	25.2 ± 1.6 ^bc^	20.0 ± 1.7 ^d^	(0.6)	24.8 ± 1.0 ^c^
*Total*	2639.3 ± 127.4 ^a^	2281.1 ± 21.3 ^b^	1785.0 ± 127.5 ^c^	1269.8 ± 50.7 ^d^	(0.5)	1944.2 ± 87.3 ^c^

Results are reported as mean ± SD (*n* = 3). Mean values within rows followed by different superscript letters (a, b, c, and d) are significantly different when subjected to Tukey’s test (*p* < 0.05). ND—non-digested; OP—oral phase; GP—gastric phase; IP—intestinal phase; CP—colonic phase; n.d.—non-detected. ^†^ Values in parenthesis indicate the intestinal phenolic bioaccessibility index. Values > 1 indicate high bioaccessibility, whereas values < 1 indicate low bioaccessibility.

**Table 2:** Total phenolic content and antioxidant capacity in digested and non-digested fractions of cocoa shell flour (CSF) and digested fractions of cocoa shell extract (CSE) throughout the different phases of the in vitro digestion.

	Digestion Phase	CSF				CSE
		Digested Fraction	Non-Digested Fraction			Digested Fraction
			Free	Bound	Total	
TPC (mg GAE g^−1^)	Raw Material	–	19.7 ± 0.9 ^a^	14.5 ± 0.3 ^a^	34.2 ± 1.1 ^a^	46.3 ± 2.5 ^c^
	Oral Phase	3.1 ± 0.3 ^c^	18.0 ± 0.6 ^b^	14.2 ± 0.6 ^a^	32.2 ± 1.2 ^b^	47.2 ± 2.0 ^bc^
	Gastric Phase	2.4 ± 0.3 ^d^	15.1 ± 0.5 ^c^	13.5 ± 0.3 ^a^	28.6 ± 0.8 ^c^	50.1 ± 1.4 ^b^
	Intestinal Phase	7.7 ± 0.4 ^a^	10.5 ± 0.5 ^d^	13.9 ± 1.3 ^a^	24.4 ± 1.3 ^d^	58.0 ± 1.8 ^a^
	Colonic Phase	6.6 ± 0.5 ^b^	14.7 ± 0.5 ^c^	10.1 ± 0.5 ^b^	24.8 ± 1.1 ^d^	58.1 ± 2.2 ^a^
ABTS (mg TE g^−1^)	Raw Material	–	38.6 ± 0.7 ^a^	34.8 ± 1.0 ^ab^	73.4 ± 1.7 ^a^	85.0 ± 1.7 ^d^
	Oral Phase	2.0 ± 1.0 ^d^	37.4 ± 0.4 ^a^	34.3 ± 1.1 ^b^	71.7 ± 1.5 ^a^	88.4 ± 2.1 ^d^
	Gastric Phase	4.0 ± 0.3 ^c^	31.9 ± 1.3 ^c^	34.2 ± 1.3 ^b^	66.1 ± 2.6 ^b^	96.8 ± 2.1 ^c^
	Intestinal Phase	30.9 ± 0.7 ^b^	25.6 ± 1.7 ^d^	36.2 ± 1.3 ^a^	61.8 ± 3.0 ^c^	633.0 ± 10.4 ^a^
	Colonic Phase	38.9 ± 0.7 ^a^	33.9 ± 0.6 ^b^	25.8 ± 0.8 ^c^	59.7 ± 1.4 ^d^	601.4 ± 2.8 ^b^
FRAP (mmol TE g^−1^)	Raw Material	–	48.9 ± 2.9 ^a^	27.5 ± 1.8 ^d^	76.4 ± 4.8 ^b^	23.5 ± 1.6 ^a^
	Oral Phase	2.0 ± 0.2 ^d^	47.6 ± 1.7 ^a^	30.2 ± 1.4 ^c^	77.8 ± 3.0 ^ab^	22.4 ± 1.8 ^a^
	Gastric Phase	13.8 ± 0.6 ^c^	42.2 ± 1.2 ^b^	37.8 ± 1.5 ^b^	80.0 ± 2.7 ^a^	20.5 ± 0.8 ^b^
	Intestinal Phase	26.8 ± 1.6 ^a^	27.1 ± 1.5 ^d^	40.3 ± 1.6 ^a^	67.4 ± 3.1 ^c^	16.3 ± 1.1 ^c^
	Colonic Phase	22.5 ± 1.3 ^b^	35.9 ± 1.0 ^c^	24.6 ± 0.6 ^e^	60.5 ± 1.6 ^d^	12.0 ± 0.8 ^d^

Results are reported as mean ± SD (*n* = 3). Mean values within rows followed by different superscript letters (a, b, c, d, and e) are significantly different when subjected to Tukey’s test (*p* < 0.05). TPC—Total Phenolic Content; GAE—gallic acid equivalent; TE—Trolox equivalent.

## Data Availability

Data are contained within the article or Supplementary Material section.

## Supplementary Materials

The following supporting information can be downloaded at: https://www.mdpi.com/article/10.3390/antiox12051007/s1, Table S1: Correlation coefficients between phenolic compounds and methylxanthines, and the radical scavenging, cellular antioxidant activity, and cytoprotective properties of the cocoa shell flour (CSF) and extract (CSE).
