# Supplementary material for: Radical Scavenging and Cellular Antioxidant Activity of the Cocoa Shell Phenolic Compounds after Simulated Digestion
Source: Antioxidants (Basel). 2023 Apr 26;12(5):1007. doi: 10.3390/antiox12051007 (PMC10215648; doi:10.3390/antiox12051007)
Supplement: Supplementary file 1 [file antioxidants-12-01007-s001.zip › antioxidants-2340552-supplementary.pdf]

Radical Scavenging and Cellular Antioxidant Activity of the Cocoa Shell Phenolic Compounds after Simulated Digestion

Silvia Cañas[1,2], Miguel Rebollo-Hernanz[1,2], Patricia Bermúdez-Gómez[1,2], Pilar Rodríguez-Rodríguez,[3] Cheyenne Braojos[1,2], Alicia Gil-Ramirez[1,2], Vanesa Benítez[1,2], Yolanda Aguilera[1,2], María A. Martín-Cabrejas[1,2*]

[1] Department of Agricultural Chemistry and Food Science, Faculty of Science, C/ Francisco Tomás y Valiente, 7. Universidad Autónoma de Madrid, 28049, Madrid, Spain.

[2] Institute of Food Science Research (CIAL, UAM-CSIC). C/ Nicolás Cabrera, 9. Universidad Autónoma de Madrid, 28049, Madrid, Spain.

[3] Department of Physiology, Faculty of Medicine, Universidad Autónoma de Madrid, C/Arzobispo Morcillo 2, 28029 Madrid, Spain

* Correspondence: maria.martin@uam.es

Supplementary Table S1. Correlation coefficients between phenolic compounds and methylxanthines, and the radical scavenging, cellular antioxidant activity, and cytoprotective properties of the cocoa shell flour (CSF) and extract (CSE).

**Compounds**	**TPC**	**ABTS**	**FRAP**	**$O_2^{\bullet -}$**	**H_2O_2**	**NO**	**$ONOO^-$**	**V-IEC-6**	**V-HepG2**	**ROS IEC-6**	**ROS HepG2**
**Cocoa shell flour**											
Hydroxybenzoic acids											
Gallic acid	0.811	0.963^{*}	0.725	0.262	0.832	−0.414	0.985^{**}	0.689	0.682	0.965^{*}	0.384
Protocatechuic acid	0.433	0.738	0.559	−0.202	0.478	−0.645	0.925^{*}	0.405	0.631	0.700	0.074
Total	0.674	0.899	0.681	0.064	0.706	−0.535	0.994^{**}	0.591	0.687	0.883	0.264
N-phenylpropenoyl-L-amino acids											
N-Coumaroyl-L-aspartate *cis*	0.581	0.238	0.373	0.580	0.543	0.249	−0.173	0.573	0.194	0.198	0.757
N-Coumaroyl-L-aspartate *trans*	0.358	0.008	0.292	0.316	0.327	0.152	−0.414	0.462	0.161	−0.094	0.730
N-Coumaroyl-L-tyrosine	0.979^{*}	0.984^{**}	0.884	0.372	0.988^{**}	−0.388	0.818	0.915^{*}	0.788	0.930^{*}	0.725
N-Caffeoyl-L-DOPA *cis*	−0.393	−0.056	−0.359	−0.271	−0.366	−0.075	0.371	−0.517	−0.236	0.065	−0.776
Total	0.687	0.391	0.592	0.418	0.664	−0.018	−0.033	0.749	0.442	0.285	0.903^{*}
Flavan-3-ols											
(+)-Catechin	0.341	0.663	0.554	−0.359	0.393	−0.742	0.856	0.373	0.657	0.589	0.066
(−)-Epicatechin	−0.195	−0.485	−0.064	−0.132	−0.214	0.105	−0.799	0.030	−0.093	−0.624	0.387
Total	0.342	0.651	0.616	−0.447	0.398	−0.830	0.809	0.428	0.731	0.537	0.151
Flavonols											
Quercetin 3-*O*-glucoside	-0.979^{*}	-0.984^{**}	−0.884	−0.372	-0.988^{**}	0.388	−0.818	-0.915^{*}	−0.788	-0.930^{*}	−0.725
Quercetin 3-*O*-arabinoside	-0.979^{*}	-0.984^{**}	−0.884	−0.372	-0.988^{**}	0.388	−0.818	-0.915^{*}	−0.788	-0.930^{*}	−0.725
Total	-0.979^{*}	-0.984^{**}	−0.884	−0.372	-0.988^{**}	0.388	−0.818	-0.915^{*}	−0.788	-0.930^{*}	−0.725
Methylxanthines											
Theobromine	0.765	0.892	0.955^{*}	−0.152	0.804	−0.801	0.798	0.868	0.971^{*}	0.727	0.679
Caffeine	0.777	0.871	0.979^{*}	−0.155	0.815	−0.804	0.735	0.905^{*}	0.989^{**}	0.688	0.745
Total	0.769	0.888	0.961^{*}	−0.153	0.808	−0.803	0.785	0.770	0.976^{*}	0.719	0.696
**Cocoa shell extract**											

Hydroxybenzoic acids											
Gallic acid	0.013	0.138	−0.270	0.369	0.606	−0.514	0.548	−0.302	−0.408	−0.259	−0.915*
Protocatechuic acid	−0.393	−0.194	0.405	0.969*	−0.250	0.210	−0.323	−0.014	−0.591	−0.405	−0.244
Total	−0.087	0.067	−0.125	0.549	0.444	−0.377	0.377	−0.256	−0.488	−0.317	−0.825
N-phenylpropenoyl-L-amino acids											
N-Coumaroyl-L-aspartate *cis*	−0.963*	−0.997**	0.899	0.277	−0.643	0.855	−0.105	−0.732	0.690	0.799	0.130
N-Coumaroyl-L-aspartate *trans*	−0.317	−0.331	−0.060	−0.084	0.535	−0.250	0.869	−0.850	0.380	0.500	−0.889
N-Coumaroyl-L-tyrosine	−0.810	−0.851	0.923*	0.257	−0.899	0.982**	−0.501	−0.343	0.567	0.609	0.576
N-Caffeoyl-L-aspartate	−0.997**	−0.991**	0.925*	0.445	−0.635	0.838	−0.128	−0.726	0.552	0.696	0.042
N-Caffeoyl-L-DOPA *cis*	−0.783	−0.863	0.849	0.071	−0.795	0.921*	−0.339	−0.448	0.727	0.750	0.517
N-Caffeoyl-L-DOPA *trans*	−0.989**	−0.998**	0.919*	0.385	−0.641	0.848	−0.120	−0.731	0.605	0.737	0.075
Total	−0.988**	−0.998**	0.895	0.367	−0.592	0.813	−0.060	−0.772	0.620	0.756	0.016
Flavan-3-ols											
(+)-Catechin	0.364	0.245	−0.650	−0.765	0.839	−0.668	0.940*	−0.390	0.393	0.333	−0.623
(−)-Epicatechin	0.512	0.431	−0.786	−0.678	0.947*	−0.826	0.922*	−0.239	0.154	0.100	−0.698
Total	0.406	0.295	−0.690	−0.749	0.872	−0.713	0.940*	−0.351	0.333	0.274	−0.645
Flavonols											
Quercetin 3-*O*-glucoside	−0.987**	−0.998***	0.918*	0.376	−0.641	0.849	−0.119	−0.732	0.613	0.743	0.080
Quercetin 3-*O*-arabinoside	−0.977*	−0.999***	0.910*	0.327	−0.643	0.853	−0.112	−0.733	0.652	0.772	0.105
Total	−0.977*	−0.999***	0.910*	0.327	−0.643	0.853	−0.112	−0.733	0.652	0.772	0.105
Flavones											
Apigenin-6,8-di-C-glucoside	−0.786	−0.812	0.932*	0.323	−0.941*	0.993**	−0.589	−0.257	0.478	0.520	0.626
Methylxanthines											
Theobromine	−0.667	−0.616	0.363	0.369	0.133	0.133	0.520	−0.872	0.279	0.471	−0.735
Caffeine	−0.785	−0.759	0.503	0.331	−0.018	0.302	0.450	−0.933*	0.439	0.621	−0.600
Total	−0.668	−0.617	0.364	0.368	0.132	0.134	0.520	−0.873	0.280	0.472	−0.734

TPC: Total Phenolic Content; ABTS: ABTS antioxidant capacity; FRAP: FRAP antioxidant capacity; $O^{2\bullet -}$: $O^{2\bullet -}$ scavenging; H_2O_2: H_2O_2 scavenging; NO: NO scavenging; $ONOO^{-}$: $ONOO^{-}$ scavenging; V-IEC-6: viability-IEC-6; V-HepG2: viability-HepG2; ROS IEC-6: reactive oxygen species IEC-6; ROS HepG2: reactive oxygen species HepG2.
